# α-Conotoxin TxIB Inhibits Development of Morphine-Induced Conditioned Place Preference in Mice *via* Blocking α6β2* Nicotinic Acetylcholine Receptors

**DOI:** 10.3389/fphar.2021.772990

**Published:** 2021-12-03

**Authors:** Xiaodan Li, Jian Xiong, Baojian Zhang, Dongting Zhangsun, Sulan Luo

**Affiliations:** ^1^ Key Laboratory of Tropical Biological Resources of Ministry of Education, School of Pharmaceutical Sciences, Hainan University, Haikou, China; ^2^ Medical School, Guangxi University, Nanning, China

**Keywords:** morphine addiction, α6β2∗ nAChRs, α-conotoxin TxIB, CPP, behavioral changes

## Abstract

Morphine, the main component of opium, is a commonly used analgesic in clinical practice, but its abuse potential limits its clinical application. Nicotinic acetylcholine receptors (nAChRs) in the mesolimbic circuitry play an important role in the rewarding effects of abused drugs. Previous studies have showed that α6β2* (* designated other subunits) nAChRs are mainly distributed in dopaminergic neurons in the midbrain area, which regulates the release of dopamine. So α6β2* nAChRs are regarded as a new target to treat drug abuse. α-Conotoxin TxIB was discovered in our lab, which is the most selective ligand to inhibit α6β2* nAChRs only. Antagonists of α6β2* nAChRs decreased nicotine, cocaine, and ethanol rewarding effects previously. However, their role in morphine addiction has not been reported so far. Thus, it is worth evaluating the effect of α-conotoxin TxIB on the morphine-induced conditioned place preference (CPP) and its behavioral changes in mice. Our results showed that TxIB inhibited expression and acquisition of morphine-induced CPP and did not produce a rewarding effect by itself. Moreover, repeated injections of TxIB have no effect on learning, memory, locomotor activity, and anxiety-like behavior. Therefore, blocking α6/α3β2β3 nAChRs inhibits the development of morphine-induced CPP. α-Conotoxin TxIB may be a potentially useful compound to mitigate the acquisition and/or retention of drug-context associations.

## 1 Introduction

Morphine was usually used as a therapeutic drug for chronic pain and mental disorders caused by traumatic events (Holbrook et al., 2010). However, repeated exposures to morphine treatment were likely to develop drug addiction, and the compulsive drug-seeking behavior and relapse posed the obstacle in overcoming addiction. It was known that different adaptations in opioid-sensitive neurons and opioid receptors constitute morphine dependence, but there were also other systems participating in morphine addiction (Christie, 2008; Luo et al., 2012). Cholinergic signaling *via* the nicotinic acetylcholine receptors (nAChRs) is involved in the regulation of abused drugs which is the dopamine projection from ventral tegmental area (VTA) to nucleus accumbens (NAc) and medial prefrontal cortex (Nestler, 2004). The activation of dopamine neurons projected from VTA to NAc leads to the outflow of dopamine in NAc, which is important for the initiation of reward learning and addiction process.

There are several nAChR subtypes expressed on dopaminergic neurons, including *α*4, *α*5, *α*6, *β*2, and *β*3 subunits (Grady et al., 2007). Expression of the *α*6*β*2* nAChRs are largely limited to dopaminergic neurons in the mesolimbic pathway, mediating behavioral motivational responses to reward stimuli through dopaminergic neurotransmission (Champtiaux et al., 2002; Yang et al., 2009). Compared with wild-type mice, nicotine induces CPP but at higher doses in α6 nAChR KO transgenic mice. Also, these mice do not exhibit cocaine-induced CPP (Sanjakdar et al., 2015), but they express CPP following a lower dose of ethanol (Steffensen et al., 2018). The *α*6*β*2* nAChR antagonists, *α*-conotoxin MII [H9A; L15A] and r-bPiDI, decreased nicotine self-administration and CPP (Jackson et al., 2009; Beckmann et al., 2015). Besides, *α*-conotoxin MII [H9A; L15A] blocked cocaine-induced CPP (Sanjakdar et al., 2015). Despite many studies showing that *α*6*β*2* nAChR antagonists decrease nicotine, cocaine, and ethanol rewarding effects, their role in morphine addiction has not been reported so far.

Our team reported a novel α-conotoxin TxIB from *Conus textile*, which is a strong antagonist specifically targeting *α*6/*α*3*β*2*β*3 nAChR with an IC_50_ of 28 nM and has almost no blocking activity on other subtypes of nAChRs expressed in *Xenopus laevis oocytes*. Nuclear magnetic resonance and structural analysis showed that the folding mode of TxIB is similar to other conotoxins, but the hydrophobic patch is smaller, resulting in higher selectivity of TxIB (Luo et al., 2013). *α*-Conotoxin TxIB contains 16 amino acids and four cysteine residues, whose sequence is GCCSDPPCRNKHPDLC-amide (Luo et al., 2013). To extensively study the pharmacological activity, we optimized the synthesis and oxidative folding conditions of TxIB to increase its yield (Wu et al., 2013). *α*-Conotoxin TxIB is a small peptide with shortcomings such as poor stability, short half-life, and poor bioavailability. In order to improve these, it has been cyclized and structurally modified for future applications (Li et al., 2020; Zhang et al., 2021). Previous studies evaluated the anti-nicotine addiction activity of TxIB by establishing a nicotine-induced CPP model, and the results showed that TxIB has obvious inhibitory effect on the establishment and relapse of nicotine-induced CPP (You et al., 2019).

The aim of the present study was to evaluate the anti-addiction effect of *α*-conotoxin TxIB, a specific antagonist *α*6/*α*3*β*2*β*3 nAChRs, on the morphine-induced conditioned place preference (CPP) in mice. Behavioral changes induced by *α*-conotoxin TxIB in various mouse models were evaluated systematically.

## 2 Materials and Methods

### 2.1 Chemical Synthesis of *α*-Conotoxin TxIB

*α*-Conotoxin TxIB is obtained from *Conus textile* by gene cloning. The linear peptide is synthesized by GL Biochem (Shanghai, China) using Fmoc chemistry, and the cysteine side chain was protected by acetamidomethyl (Acm) and triphenylmethyl (Trt). The linear peptide underwent a two-step oxidation method to remove the protective groups and form two disulfide bonds as previously described (Wu et al., 2013). In the first step, the linear peptide was mixed with 20 nM potassium ferricyanide and 0.1 M Tris with pH 7.5 at room temperature for 45 min to form the first disulfide bond. In the second step, the monocyclic peptide was kept in 1 mM iodine solution with 24% acetonitrile and 3% TFA aqueous for 10 min resulting in the formation of TxIB. And finally, the purity and structure of TxIB identified using RP-HPLC and LCMS-IT-TOF mass spectrometry (Shimadzu, Kyoto, Japan).

### 2.2 Animals

Male C57BL/6J mice (20–22 g), 6 weeks old, bought from SJA Laboratory Animal Co., Ltd. (Changsha, China) were housed in plastic cages with nesting material and had free access to food (standard mouse chow) and water. Temperature (23 ± 1°C) and humidity (50–60%) of animal laboratory were kept in a consistency. The experiments were conducted in the light period during 12-h light–dark cycle (8:00 am–8:00 pm). Animals were handled for 3–4 days to adjust to laboratory conditions before any experiment. The International Association for The Study of Pain (IASP) guidelines on the use of awake animals were followed in this study, and efforts were also made to minimize the number and discomfort of animals. This study was approved by the Hainan University Institutional Animal Use and Care Committee (HNUAUCC-2021-00056).

### 2.3 Lateral Ventricle Cannula Implantation and Infusions

For cannulation surgeries, mice were anesthetized with isophorone using Mice and Rat Animal Anesthesia Machine (RWD, Shenzhen, China). Isoflurane is a colorless clear liquid of diethyl ether with the molecular formula C_3_H_2_CIF_5O_. It is one kind of anesthetic used for surgery. The concentrations for induction and maintenance of anesthesia were 3 and 1.5%, respectively. All surgeries were performed using aseptic procedures. An incision was made to expose the skull of the mouse. The mouse’s head was leveled using the stereotaxic apparatus. Then the 26-gauge guide cannula was implanted into the lateral ventricle of the brain (coordinates relative to bregma: AP =−0.6 mm, ML = + 1.3 mm, DV =−2.0 mm) and reinforced by dental glue and dental cement. The cannula was secured with a dust cap to prevent post-surgical infection and obstruction. Penicillin powder was applied to the wound to prevent infection. After completion of surgeries, the mice were returned to clean home cages, each for one cage. At the end of the experiment, the brains were harvested to verify cannula placement.

### 2.4 CPP Paradigm

The CPP apparatus consisted of two side compartments and one central compartment that could be separated by guillotine doors with an auto-monitoring system obtained from AniLab, Ningbo, China. Two side compartments (left and right) had the same size (17.38 × 13.5 × 15 cm) and different wall colors and different floor textures. The left compartment had white walls and round hole mesh floor. The right compartment had black walls and grid rod floor. The center compartment (9.8 × 13.5 × 15 cm) was just a protruded tunnel connecting two main chambers. All apparatus were placed in a soundproof room to avoid affecting by noise.

#### 2.4.1 Preconditioning

Before the experiment, all mice were placed in the center compartment without a drug injection and allowed to freely explore all of the apparatus for 15 min for once per day for 2 days (days 1–2). On day 3, the initial baseline preference was evaluated by recording the time that the mice spent on each box, which was used to determine the CPP scores in experimental mice after morphine administration. Animals who had natural preferences toward any compartment (more than 540 s) were excluded from the study.

#### 2.4.2 Conditioning

The place conditioned training consisted of 40-min sessions, twice per day (6 h apart) for 4 consecutive days (days 4–7). Between 8:30 and 12:00, each animal was injected with morphine (5 mg/kg, s. c.) purchased from the China Drug Inspection Center and immediately confined to the drug-paired compartment of the apparatus for 40 min. The mice were returned to their home cages immediately after the session. Between 14:30 and 18:00, each animal was confined to a non-drug–paired compartment following subcutaneous injection of saline. The control group was only injected with saline twice per day in both compartments with the door closed. *α*-Conotoxin TxIB or saline was injected (i.c.v.) 50 min before injection of morphine or saline (s.c.) to explore its effect on the development of morphine-induced CPP.

#### 2.4.3 Post-Conditioning Test

24 h after the last training session, the mice were placed into the CPP apparatus with the guillotine doors removed and allowed to freely explore the compartments of the apparatus for 15 min. The amount of time that mice spent in each compartment was recorded. CPP scores were calculated as the time spent in the drug-paired compartment after training (or administration) minus the time spent in the drug-paired compartment at preconditioning.

### 2.5 Morris Water Maze Test

The Morris water maze (MWM) testing was conducted to examine learning and memory in subsequent behaviors according to previous methods (Vorhees and Williams, 2006). The Morris water maze equipment consists of a maze, platform, water, and tracking system. The maze is a round pool constructed out of gray polypropylene plastic, 110 cm in diameter and 50 cm in height. The platform is circular transparent Plexiglas 8.5 cm in diameter with round grooves. Water temperature was maintained at 24–25°C by a heating device at the bottom of the pool. The tracking and analysis systems use Smart 3.0 (Panlab, United States). The Morris water maze experiment consisted of three different trials: visible platform testing, hidden platform testing, and probe trial. At the end of each trial, the mouse was wiped out with a dry towel and warmed with warm lamp lighting for 5 min before returning to the home cage.

#### 2.5.1 Visible Platform (Cued) Testing

The platform was elevated above the surface of water by 1 cm and a little red flag was located on the platform to guide the mice onto the platform. Each mouse was placed in a different starting location with nose facing the wall to reach the corresponding platform. The tests were done to examine the ability of swimming, vision, and the motivation to escape. The time taken to reach the platform was recorded. If the mice did not reach the platform within 60 s, then they were guided to the platform, and the time was recorded as 60 s. The mice were allowed to or guided to stay on the platform for 20 s to orientate the distal visual cues. There were no significant differences in the latency period of escape between different groups.

#### 2.5.2 Hidden Platform (Place) Testing

The platform was placed under the surface of water by 1 cm and white non-toxic tempera paint (Titanium dioxide) was placed into water to camouflage the platform. Four trials per day for 5 consecutive days were performed to let mice learn to use cues to find the hidden platform. The mice found or guided to the platform were left on the platform for 20 s during the inter-trial interval. Each trial lasted for a maximum of 60 s. The amount of time that mice spent to find the platform was recorded by an auto-tracking system.

#### 2.5.3 Probe Trial

The probe trial was conducted to examine spatial reference memory. 24 hours after the hidden platform test, the platform was removed. The mice were placed in the maze at a novel start position with the nose facing the tank wall and allowed to explore the maze for 60 s, then removed from the pool. The percent of time or percent distance in the target quadrant, latency to first target-site, number of platform-site crossovers were measured by an auto-tracking system.

### 2.6 Locomotor Activity

The locomotor activity was registered with a video tracking software (Smart 3.0, Panlab, United States) in a sound-attenuated, ventilated, and dimly lit plastic box (40 × 40 × 35 cm) for 60 min. Before the start of the experiment, all mice were adapted to the spontaneous activity box for 5 min on the first day. The locomotor activity was recorded 30 min after intraventricular injection of TxIB or saline. The spontaneous activity boxes were cleaned with ethanol solution to avoid the influence of odor.

### 2.7 Elevated Plus Maze Test

The elevated plus maze (EPM) was utilized to evaluate the anti-anxiety effects of pharmacological agents based on the mice’s unconditioned fear of open, elevated, and unprotected spaces. The EPM is composed of two open arms (32 × 8.5 cm) and two closed arms of the same size. All arms were connected by a common central area (8.5 × 8.5 cm). The maze was elevated above the floor at a height of 40 cm. Each mouse was placed in the central area of the maze with its nose facing an open arm and allowed to freely explore the maze for 5 min. The activity of mice in EPM was recorded with a video tracking software (Smart 3.0, Panlab, United States) after the locomotor activity test.

### 2.8 Dosing Regimen

**FIGURE 1:**
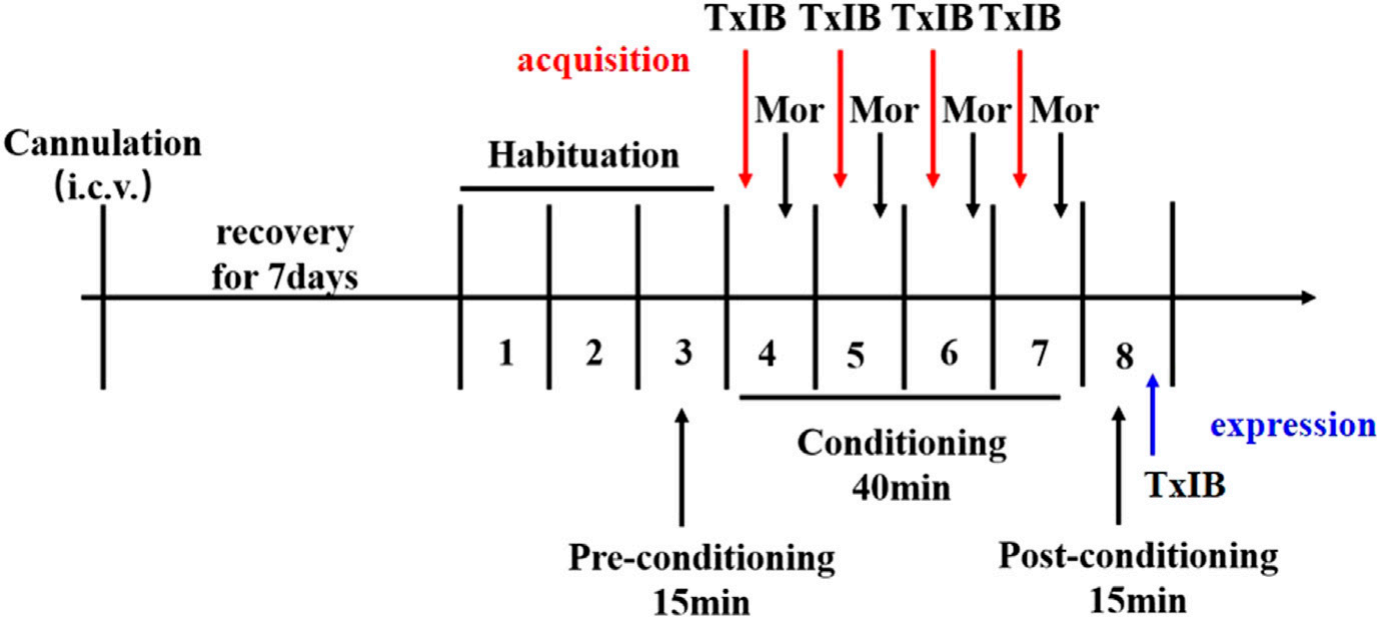
Schematic diagram of the experiment of morphine-induced CPP.

For lateral ventricle infusions, α-Conotoxin TxIB or saline was delivered under 1.25 μL/min through Hamilton 1700 and an auto-micro infusion pump (KD Scientific, MA, United States). After drug delivery, the injector was left in place for 2 more minutes to avoid reflux of the drug. To examine the effects of blocking *α*6/*α*3*β*2*β*3 nAChRs on morphine-induced behavior, *α*-conotoxin TxIB was injected during morphine-induced CPP expression and acquisition. For the expression, the successfully modeled CPP mice injected with different doses of TxIB (0, 0.1, 1, and 10 nmol, i.c.v. *n* = 10–12/group) 90 min prior to the placement of mice into the CPP center chamber for testing. For the acquisition, separate groups of animals received pretreatment with TxIB (0, 0.1, 1, and 10 nmol, i.c.v. *n* = 8–10/group) 50 min prior to receiving an injection of 5 mg/kg morphine during the conditionings. Immediately following the morphine injection, animals were placed into their drug-paired compartment. The experimental process is shown in Figure 1.

**FIGURE 2:**
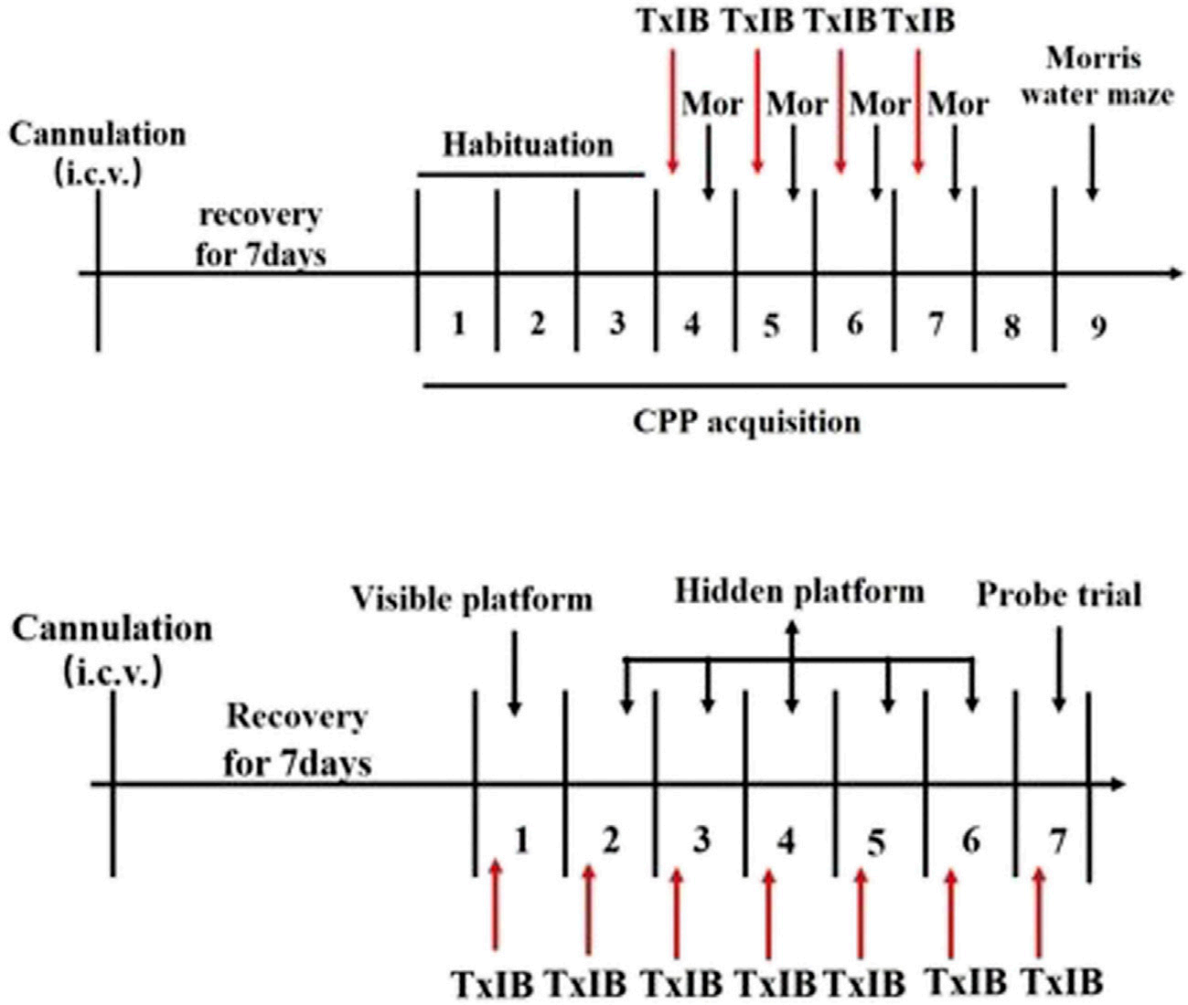
Schematic diagram of the experiment of Morris water maze.

To detect the effects of blocking *α*6/*α*3*β*2*β*3 nAChRs on learning and memory behavior, the Morris water maze experiment was performed. After testing the effect of TxIB on the acquisition of morphine-induced CPP in mice, the Morris water maze (MWM) testing was conducted to examine learning and memory in subsequent behaviors. Besides, the C57BL/6J mice received pretreatment with TxIB (0, 1 nmol, i.c.v. *n* = 7/group) 90 min prior to Morris water maze tests for continuous 7 days to detect the direct effects of TxIB on learning and memory behaviors. The experimental procedure is shown in Figure 2.

**FIGURE 3:**
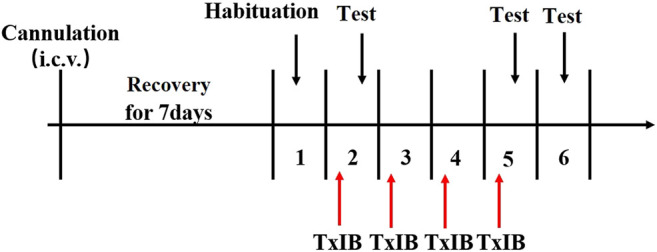
Schematic diagram of the experiment of locomotor activity and elevated plus maze test.

To detect the effects of blocking *α*6/*α*3*β*2*β*3 nAChRs on locomotor activities and anxiety-like behaviors, separate groups of animals received pretreatment with TxIB (0, 0.1, 1, and 10 nmol, i. c.v. *n* = 8–12/group). The experimental procedure is shown in Figure 3. Acute and repeated α-conotoxin TxIB injections were used to measure changes in locomotor activity and anxiety-like behavior. For acute treatment, the mice were treated with *α*-conotoxin TxIB 30 min prior to locomotor tests and EPM tests on the day of the first injection. For repeated treatment, the mice were treated with *α*-Conotoxin TxIB for 4 days with locomotor tests and EPM tests occurring 30 min after the last α-conotoxin TxIB injection.

### 2.9 Statistical Analyses

All data are shown as mean ± SEM and analyzed using GraphPad Prism 5.0. For CPP expression and acquisition experiment, Morris space exploration experiment, locomotor activity experiment, elevated plus maze experiment, one-way analysis of variance (one-way ANOVA), and Dunnett’s multiple comparisons test were used to compare data. For the distance traveled during CPP acquisition, Morris water maze positioning and navigation experiment, two-way analysis of variance (two-way ANOVA) and Bonferroni’s post hoc method for analysis were used to compare data. *p* < 0.05 is considered a significant difference.

## 3 Results

### 3.1 *α*6/*α*3*β*2*β*3 nAChR Specific Antagonist TxIB Inhibited Expression of Morphine-Induced CPP

**FIGURE 4:**
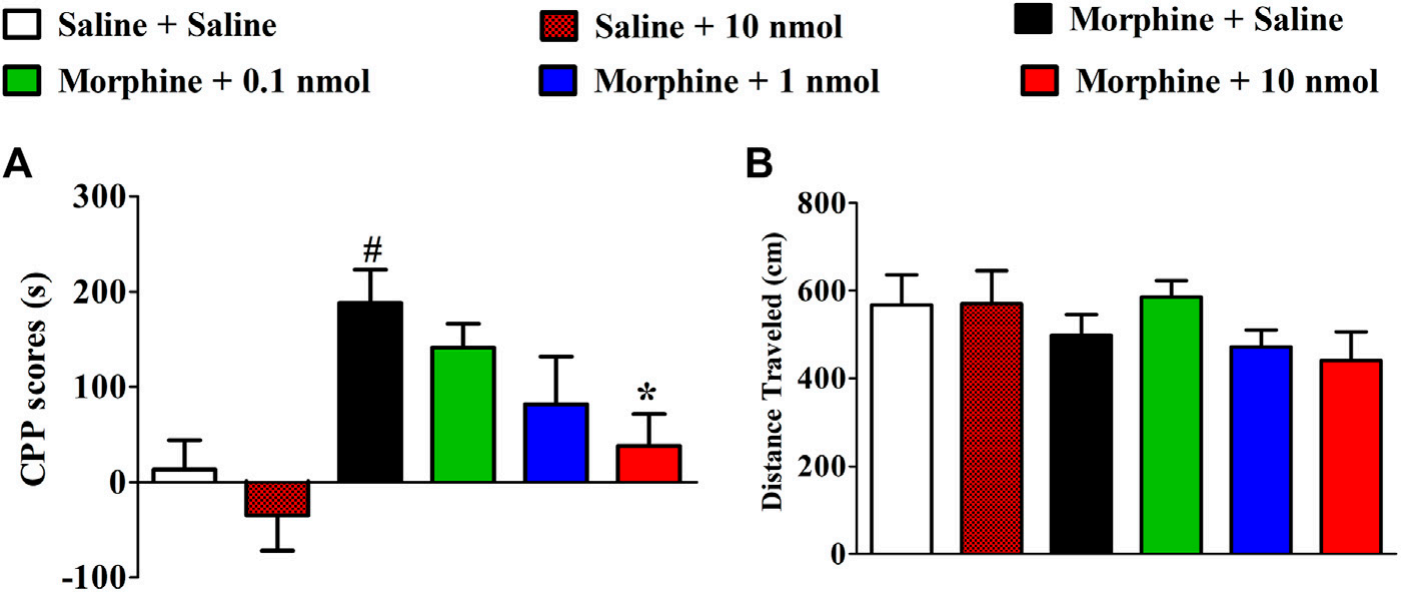
Effects of *α*-conotoxin TxIB on the expression of morphine-induced CPP. Results are expressed as mean preference scores ±SEMs for 9–12 mice. **(A)** The CPP scores of post-conditioning test, CPP scores = the time spent in drug-paired compartment after administration—preconditioning. ANOVA for the CPP scores: F_5,63_ = 4.72, *p* < 0.05; **(B)** the distance traveled on the post-conditioning test day. ANOVA for the traveled distance: F_5,63_ = 1.07, *p* = 0.38; # denotes a significant difference from the saline + saline group; * denotes a significant difference from the morphine + saline group (# = *p* < 0.05,* = *p* < 0.05).

After 4 days of conditioned training, the time spent in drug-paired compartments of mice significantly increased which were used to evaluate the effect of TxIB on the expression of morphine-induced CPP. As shown in Figure 4A, α6/α3β2β3 nAChR antagonist TxIB significantly attenuated expression of morphine CPP at dose of 10nmol/mouse (F_5,63_ = 4.72, *p* < 0.05). There was a downward trend at a lower dose of 1nmol/mouse, but it was not significant (*p* > 0.05). Single TxIB injection at the highest dose of 10 nmol/mouse did not produce a preference or aversion in saline-treated mice during the expression test. The distance traveled of post-conditioning test was monitored at the same time (Figure 4B). There was no significant difference among the groups (F_5,63_ = 1.07, *p* > 0.05).

### 3.2 *α*6/*α*3*β*2*β*3 nAChR Specific Antagonist TxIB Inhibited Acquisition of Morphine-Induced CPP

**FIGURE 5:**
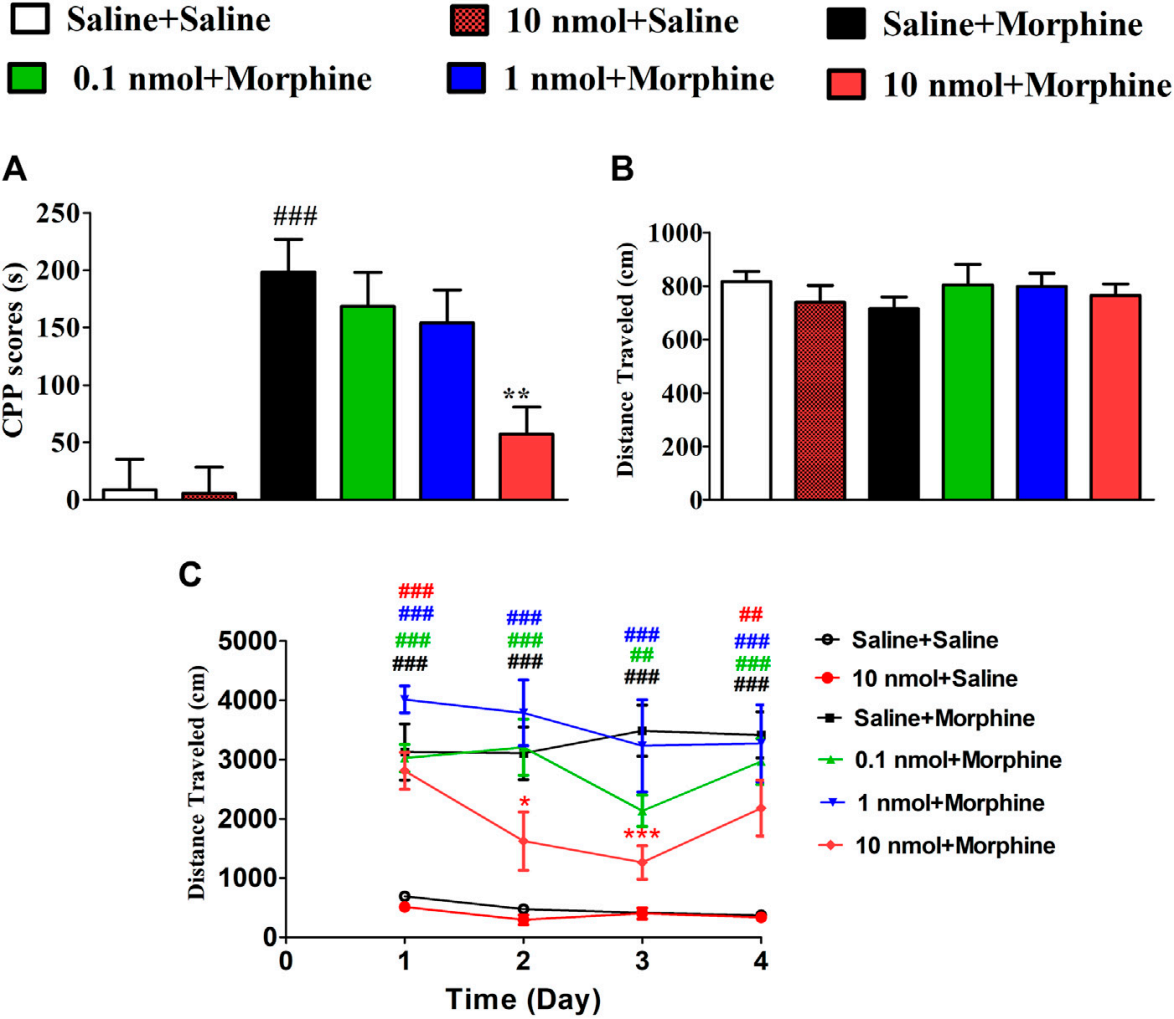
Effects of *α*-conotoxin TxIB in acquisition of morphine-induced CPP. Results are expressed as mean preference scores ±SEMs for 8–10 mice. **(A)** The CPP scores of post-conditioning test, CPP scores = the time spent in drug-paired compartment during post-conditioning test—preconditioning. ANOVA for the CPP scores: F_5,47_ = 10.07, *p* < 0.001; **(B)** the distance traveled of post-conditioning test. ANOVA for the distance traveled: F_5,47_ = 0.53, *p* = 0.75; **(C)** the distance traveled during CPP acquisition. # denotes a significant difference from the saline + saline group; * denotes a significant difference from the saline + morphine group (** = *p* < 0.01, #### = *p* < 0.001).

Mice were pretreated with different doses of TxIB to evaluate its effects on the acquisition of morphine-induced CPP. The mice pretreated with saline and conditioned with morphine exhibited a robust CPP, and the time spent in the drug-paired compartment increased from 286.4 ± 49.2 s to 484.7 ± 88.9 s. Pretreatment with TxIB dose-dependently reduced the acquisition of morphine-induced CPP (Figure 5A). Compared with the saline + morphine group, the CPP scores of TxIB 10 nmol + morphine group were significantly reduced (F_5,47_ = 10.07, *p* < 0.01). The CPP scores of TxIB 10 nmol injected 4 consecutive days (10 nmol + Saline) had no obvious difference compared with the saline + saline group, showing that TxIB itself did not produce a preference or aversion at the highest dose. The distance traveled during post-conditioning test was monitored at the same time (Figure 5B). Compared with the saline + morphine group, there was no significant difference in all doses of TxIB (F_5,47_ = 0.53, *p* > 0.05). The total distance traveled during CPP acquisition is shown in Figure 5C. Compared with the saline-conditioned mice, the morphine-conditioned groups have significant increases in the distance traveled during CPP acquisition. Compared with the saline + saline group, the distance traveled of saline + morphine increased significantly (*p* < 0.001). Continuous administration of TxIB 0.1 and 1 nmol did not reduce morphine-induced hyperlocomotion. The distance traveled of TxIB 10 nmol + morphine group significantly reduced, with a significant difference at Day 2 and Day 3.

### 3.3 *α*6/*α*3*β*2*β*3 nAChR Specific Antagonist TxIB Did Not Affect the Learning and Memory in MWM

**FIGURE 6:**
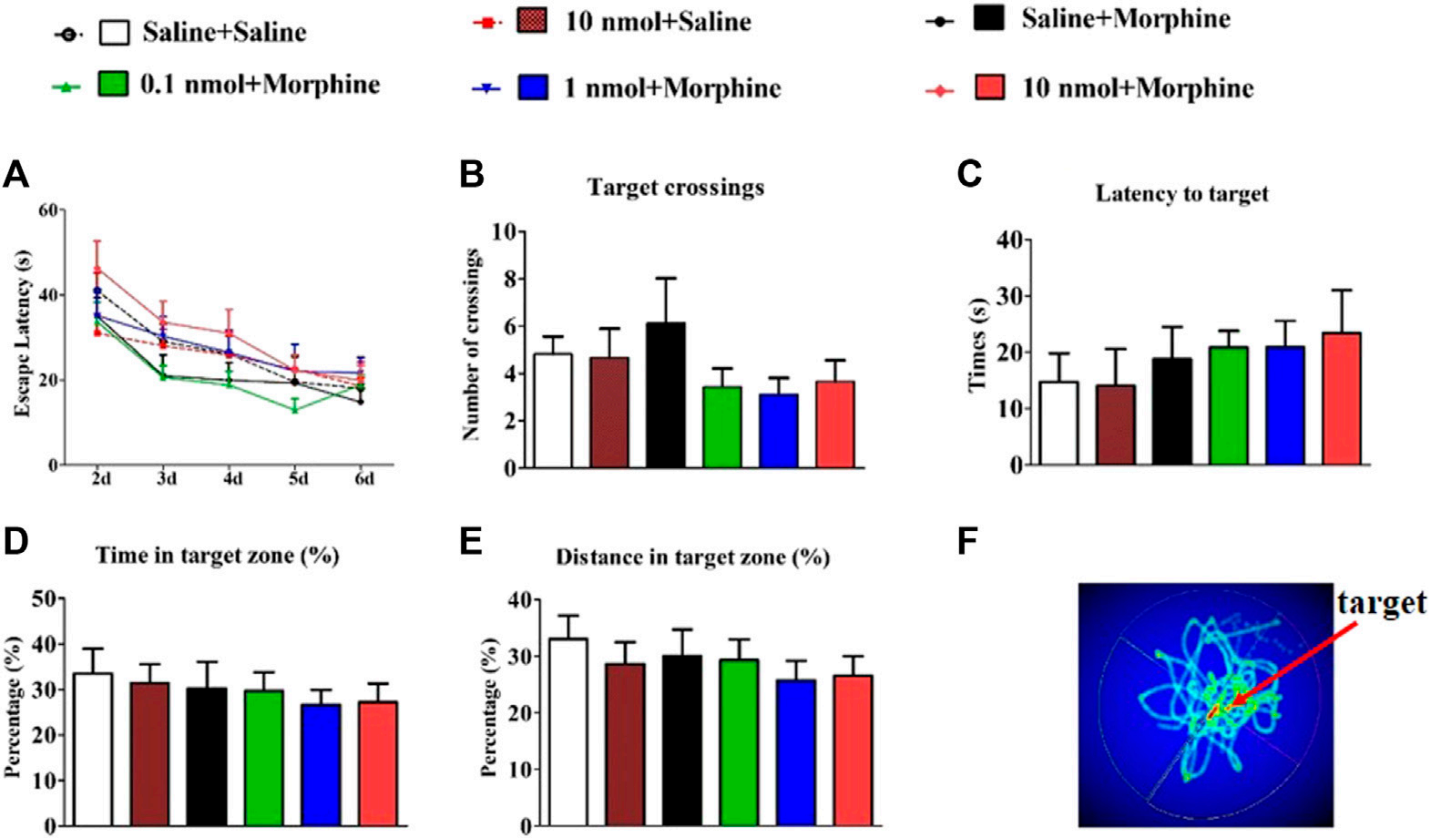
Effects of α-conotoxin TxIB in Morris water maze test. Data are presented as the mean ± SEM, n = 8–10. **(A)** The escape latency from water onto the platform during the hidden platform test. Two-way RM ANOVA for the escape latency: interaction, F_20,192_ = 0.65, *p* = 0.87; time, F_4,192_ = 24.83, *p* < 0.001; the different doses group, F_5,192_ = 1.22, *p* = 0.31. Spatial memory was assessed in a probe trial with respect to: **(B)** target crossings, **(C)** latency to target, **(D)** percent of time in target quadrant, **(E)** percent of distance in target quadrant. There was no significance among the six groups. ANOVA for spatial memory in a probe trial: target crossings, F_5,47_ = 1.22, *p* = 1.02; latency to target, F_5,47_ = 0.43, *p* = 0.82; percent of time in target quadrant, F_5,47_ = 0.33, *p* = 0.89; percent of distance in target quadrant, F_5,47_ = 0.50, *p* = 0.78. **(F)** Thermal image of mouse trajectory in Morris water maze probe trial. Mice to explore the nearby former platform position significantly more than elsewhere.

**FIGURE 7:**
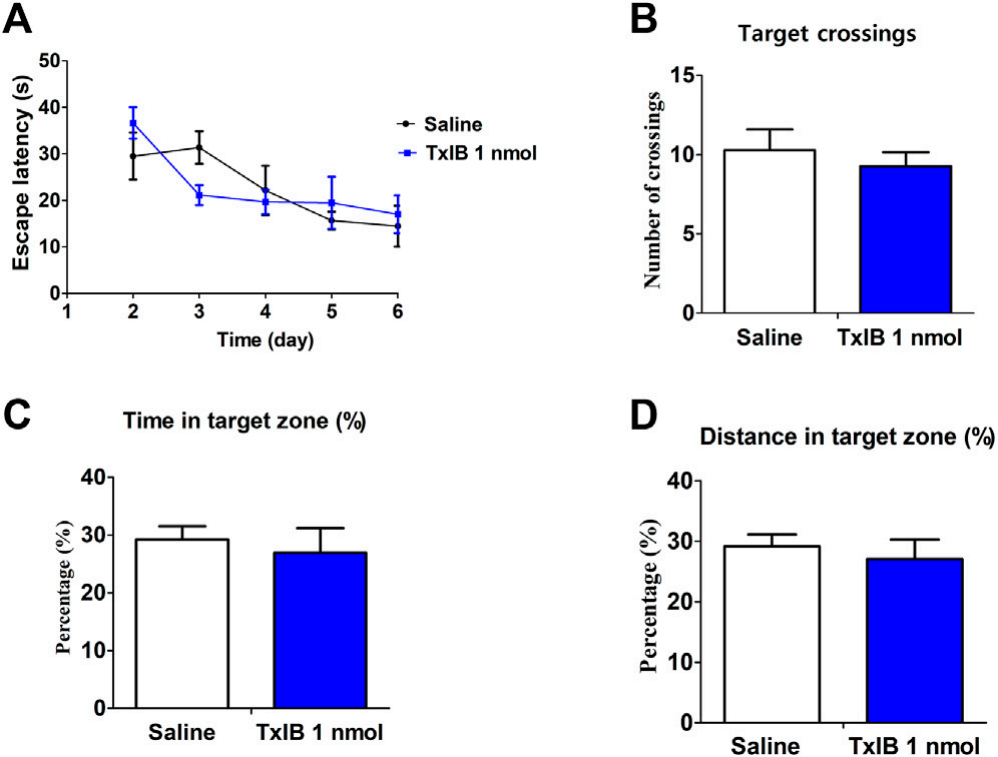
Direct effects of α-conotoxin TxIB in Morris water maze test. Data are presented as the mean ± SEM, *n* = 7. **(A)** The escape latency from water onto the platform during the hidden platform test. Spatial memory was assessed in a probe trial with respect to: **(B)** target crossings, **(C)** percent of time in target quadrant, **(D)** percent of distance in target quadrant. Compared with the saline group, there was no significance.

Since CPP procedure is to establish learned associations between reward stimuli and a specific non-reward neutral stimulus (such as the environment). After testing the effect of TxIB on the acquisition of morphine-induced CPP in mice, we further tested the effect of α6β2* nAChR antagonist on learning and memory in subsequent behaviors by Morris water maze (Figure 6). The C57BL/6J mice received pretreatment with TxIB 90 min before Morris water maze tests for continuous 7 days to detect the direct effects of TxIB on learning and memory behaviors (Figure 7). The mouse performs a visual platform test after being treated with *α*-conotoxin TxIB during morphine acquisition. During the hidden platform test, the amount of time spent on escaping from water onto the platform (escape latency) was recorded (Figure 6A). On day 6, the escape latency was significantly decreased by training compared to day 2 (F_4,192_ = 24.83, *p* < 0.001), but there was no statistical difference among all groups (F_5,192_ = 1.22, *p* > 0.05). A probe trial was conducted to examine the spatial memory after the hidden platform test. During the probe trial, the amount of target (location of the removed platform) crossings (Figure 6B), latency of first time to target (Figure 6C), time and distance in target quadrant, percentage of time (Figure 6D), and distance (Figure 6F) in target quadrant were recorded and analyzed. There is a slight difference between the groups, but not statistically significant. Continuous injection of TxIB did not directly and indirectly disrupt learning and memory in subsequent behaviors and further explained that the effect of TxIB on morphine-induced CPP is not due to memory impairment.

### 3.4 Effects of *α*6/*α*3*β*2*β*3 nAChR Specific Antagonist TxIB in Locomotor Activity

**FIGURE 8:**
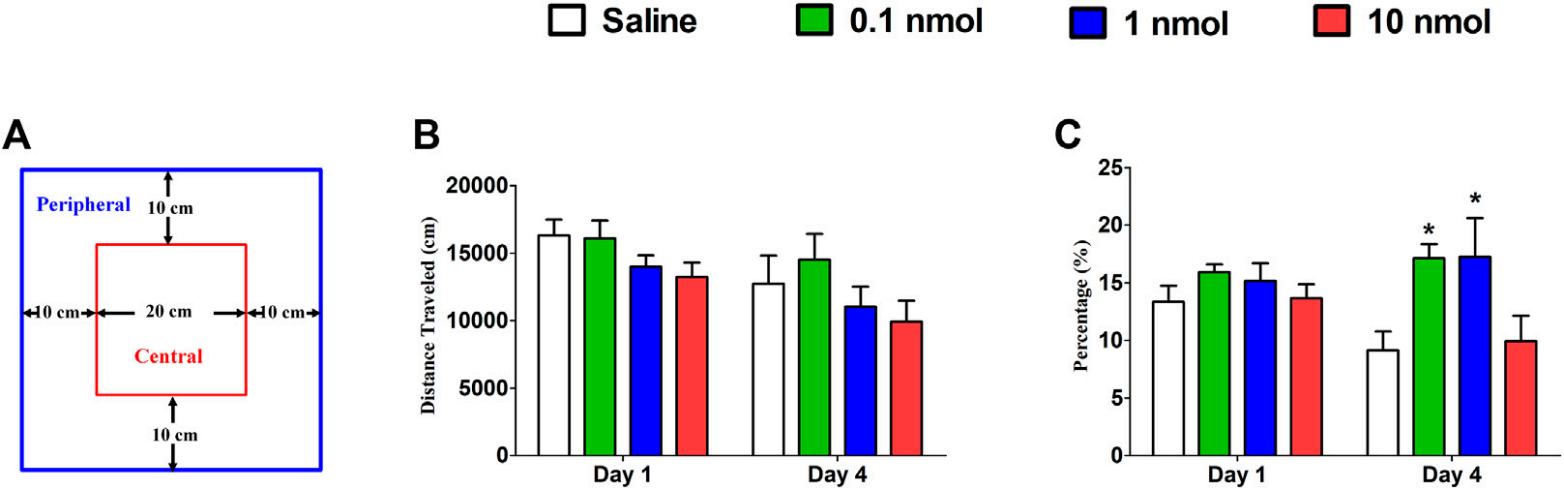
Effects of *α*-conotoxin TxIB in locomotor activity test. Data are presented as the mean ± SEM, *n* = 8–10. **(A)** Schematic diagram of the locomotor activity box. **(B)** The distance traveled of different groups on the first and fourth days. ANOVA: Day 1, F_3,34_ = 1.84, *p* = 0.16; Day 4, F_3,34_ = 1.26, *p* = 0.30. **(C)** The percentage of the distance in the central area to the total distance of different groups on the first and fourth days. ANOVA: Day 1, F_3,34_ = 0.93, *p* = 0.44; Day 4, F_3,34_ = 4.02, *p* < 0.05. Asterisks represent significant difference of the saline group (* = *p* < 0.05).

**FIGURE 9:**
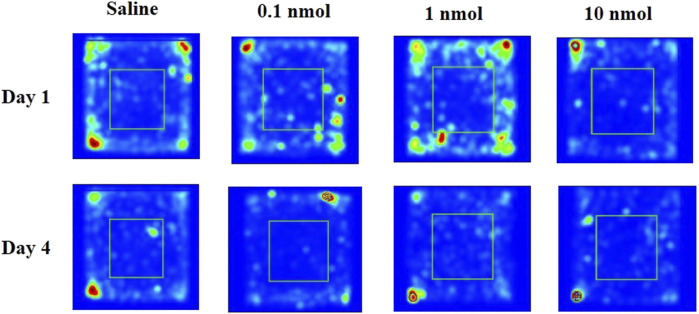
The traces of mouse injected with saline or TxIB during locomotor activity test.

A locomotor activity video analysis system is used to study the neuropsychological changes of experimental animals and various behaviors after entering the open environment. For example, when entering a new environment, mice will mainly move in the peripheral area due to fear, and the exploratory characteristics prompt them to enter the central area to further observe the resulting anxiety. When mice were treated with different doses of TxIB, it did not increase or decrease the distance traveled on the first and fourth days compared with the normal saline group (Figure 8B). These results suggest that different doses of TxIB did not affect the locomotor activity (*p* > 0.05). However, when the activity data were further analyzed, the results showed that after 4 consecutive days of injection of low and medium doses of TxIB, the percentage of the distance in the central area to the total distance significantly increased compared with the saline group and the high dose of TxIB (*p* < 0.01) (Figure 8C). The traces of mouse injected with saline or TxIB during locomotor activity test were shown in Figure 9.

### 3.5 *α*6/*α*3*β*2*β*3 nAChR Antagonist TxIB Did Not Alter Anxiety-like Behavior

**FIGURE 10:**
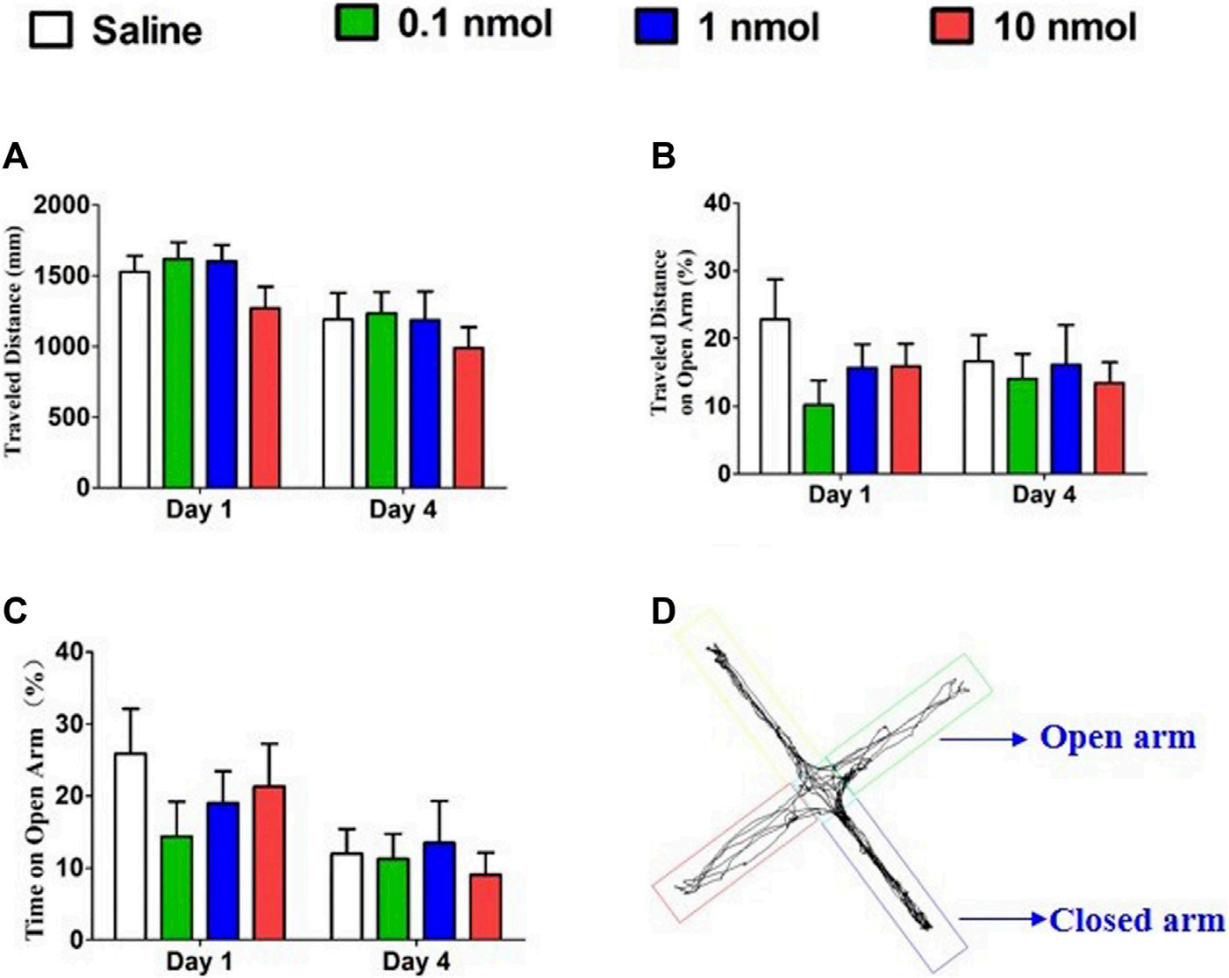
Effects of *α*-conotoxin TxIB in the elevated plus maze test. Data are presented as the mean ± SEM, *n* = 8–10. **(A)** The distance traveled by different groups on the first and fourth days. ANOVA: Day 1, F_3,32_ = 1.63, *p* = 0.20; Day 4, F_3,34_ = 0.43, *p* = 0.73. **(B)** The percentage of travel distance in the open arm of different groups on the first and fourth days. ANOVA: Day 1, F_3,32_ = 1.39, *p* = 0.26; Day 4, F_3,34_ = 0.15, *p* = 0.93. **(C)** The percentage of time spent in the open arm of different groups on the first and fourth days. ANOVA: Day 1, F_3,32_ = 0.72, *p* = 0.55; Day 4, F_3,34_ = 0.22, *p* = 0.88. **(D)** The traces of mice during elevated plus maze test.

The principle of the elevated plus maze is to assess the level of anxiety in rodents by using the conflict between the rodent’s internal motivation and natural tendency to explore new spaces. The decreased amount of time spent on the open arms can be considered as a manifestation of high anxiety. When mice were treated with different doses of TxIB, the distance traveled, percent of travel distance in the open arm, and percent time spent in the open arm were not significantly increased or decreased on the first and fourth days compared with the normal saline group (Figure 10). Therefore, injecting different doses of TxIB for 4 consecutive days did not alter the anxiety-like behavior of the mice in the elevated plus maze test.

## 4 Discussion

The “World Drug Report 2020” issued by the United Nations Office on Drugs and Crime pointed out that more than 35 million people worldwide are currently addicted to drugs (United Nations Office on Drugs and Crime, 2020). Among them, marijuana is the most commonly abused substance, but opioid drugs are the most harmful. Opioid drugs such as morphine exert their pharmacological effects by stimulating opioid receptors located in the peripheral and central nervous systems. The *μ*-opioid receptor is the key to the action of morphine and a series of studies show a strong relationship between the activation of the *μ*-opioid receptor located in the VTA and the reinforcing effects of morphine (Bodnar, 2016; Listos et al., 2019a). Generally, the stimulation of structures within the mesolimbic system is closely related to the rewarding effect of addictive substances. Although addictive drugs act on different targets, what ultimately activates is the reward circuit in the mesocorticolimbic dopaminergic system, resulting in increased dopamine release in the nucleus accumbens (Di Chiara, 2000; Chartoff et al., 2006), which determines the feeling of pleasure. *α*6*β*2* nAChRs are limitedly distributed in dopaminergic cells in the mesocorticolimbic. Previous studies have reported that *α*6/*α*3*β*2*β*3 nAChRs antagonists can inhibit drug addiction such as nicotine, alcohol, and cocaine (Brunzell et al., 2010; Kamens et al., 2017; Maggio et al., 2018; Avelar et al., 2019). However, the role of *α*6β2* nAChRs in morphine addiction has not been verified so far. Our current work verified for the first time that blocking *α*6*β*2* nAChRs inhibits the acquisition of morphine-induced CPP without affecting learning and memory in mice.

The CPP model is commonly used to evaluate the anti-addition potential of drug and reward properties of abuse drugs in rodent animals. The CPP model was successfully established by subcutaneous injection of 5 mg/kg morphine for 4 consecutive days in the current study (Shoblock et al., 2005). Single intracerebroventricular injection of TxIB can dose-dependently attenuate expression of morphine-induced CPP. Pretreatment with *α*6/*α*3*β*2*β*3 nAChR antagonist TxIB for 4 consecutive days can inhibit the acquisition of morphine-induced CPP, and the highest dose (10 nmol) completely abolished the CPP response. Currently used medication for opiate addiction, such as buprenorphine and methadone, would produce reward and reinforcement and have a possibility of abuse. Our results revealed that continuous injection of TxIB did not produce a preference or aversion in place conditioning paradigm. It has been shown in previous studies that a single intracerebroventricular injection of TxIB can inhibit the expression of nicotine-induced CPP and reduce the concentrations of dopamine and GABA in VTA, NAc, hippocampus (HIP), and prefrontal cortex (PFC) in the brain during the expression period of CPP (You et al., 2019). Notably, the effective dose in the morphine-induced CPP model was significantly higher than that in the nicotine-induced CPP model as previous work had used. The reason for this may be that morphine-activating opioid receptors cause a series of neurotransmitter changes in the reward pathway, such as acetylcholine, dopamine, and GABA thereby leading to dependence, while nicotine directly acts on nAChRs. There are other pathway and complex mechanisms participating in reward property of morphine addition pathways (Kim et al., 2016; Listos et al., 2019). Morphine selectively promotes the release of glutamate from the medial prefrontal cortex to VTA-DA neuron by eliminating the inhibition of GABA_B_ receptors in glutamatergic input from the medial prefrontal cortex (Chen et al., 2015; Yang et al., 2020). Besides, morphine-induced increase in locomotor activity relies on dopamine release within the mesocorticolimbic circuit, and the high dose of *α*-conotoxin TxIB can significantly reduce the distance traveled during morphine-induced CPP acquisition. *α*-Conotoxin TxIB inhibits the acquisition of morphine-induced CPP by suppressing the release of DA in the mesocorticolimbic possibly, but the specific mechanism needs to be further verified.

Addiction is a learning process. Morphine-induced CPP model is to establish learned associations between reward stimuli and a specific non-reward neutral stimulus (such as the environment) based on the Pavlovian classical conditioning theory. Previous studies have combined with conditioned place preference (CPP) training to research the learning and memory-related brain circuits in the process of drug dependence (Keleta and Martinez, 2012; Keyes et al., 2020). Acetylcholine is an important neurotransmitter, which can regulate various forms of neuroplasticity and contribute to learning and memory. Scopolamine, a non-selective muscarinic cholinergic antagonist, disrupted learning and memory in passive avoidance response and impaired spatial learning and memory in the Morris water maze (Entlerova et al., 2013). The DA signal in the HIP-mPFC connection is involved in morphine-associated and normal memory3, and the DA, D1R, and D2R play a role in the acquisition and retrieval of morphine-induced CPP (Wang et al., 2019). Electrophysiological studies have shown that nAChRs containing *β*2 subunits are necessary for nicotine’s ability to increase the depolarization and firing rate of dopamine neurons in VTA (Wittenberg et al., 2020). Therefore, it is necessary to study the role of *α*6*β*2* nAChRs on morphine acquisition–related memory. The *α*6/*α*3*β*2*β*3 nAChR specificity antagonist, *α*-conotoxin TxIB did not impair memory, as observed through examining spatial learning and memory in the Morris water maze in the present work.

Motor activity in the CPP paradigm was an effective sign for studying drug addiction and psychostimulant-induced psychosis. In the study, repeated injections of 5 mg/kg morphine did not increase the motor activity of the mice, which was sufficient to induce CPP addiction; at the same time, single or repeated injections of TxIB had no effect on motor activity in the CPP paradigm. Previous studies have shown that locomotor response to novelty was linked to mechanisms of addiction and stress (Hooks et al., 1991; Mandt et al., 2009). For example, animals with high responses to novelty were found to show higher predisposition to drug self-administration and higher sensitivity for natural reinforcers and stressors (Dellu et al., 1996; Pierre and Vezina, 1997). The locomotor activity of mice indicated that repeated injections of different doses of TxIB did not affect the distance traveled. However, after 4 consecutive days of injection of low and medium doses of TxIB, the percentage of the distance in the central area to the total distance significantly increased compared with the saline group, showing the potential for anti-anxiety. When entering a new environment, mice will mainly move in the peripheral area due to fear and the exploratory characteristics prompt them to enter the central area to further observe the resulting anxiety. In order to confirm the change of this anxiety in locomotor activities, we further use the elevated plus maze to verify. Therefore, the elevated plus maze was used to evaluate the effect of continuous treatment of TxIB on anxiety-like behavior in mice, and the results showed that TxIB did not cause or attenuate anxiety-like behavior. A large number of studies have shown that cholinergic neurotransmission in the brain is involved in the regulation of anxiety-like behaviors (Brioni et al., 1993; File et al., 2000; Newman et al., 2001). For example, nAChR antagonist mecamylamine produced anxiolytic effects in animal models of anxiety (Zarrindast et al., 2008). However, whether *α*6*β*2* nAChRs participate in the regulation of anxiety-like behavior remains to be further studied.

In sum, blocking *α*6*β*2*β*3 nicotinic acetylcholine receptors inhibits the development of morphine-induced CPP in mice. α-Conotoxin TxIB, *α*6/*α*3*β*2*β*3 nAChR antagonist, was a special potential anti-addiction drug without reward properties and did not affect learning, memory, locomotor activity, and anxiety-like behavior.

## Data Availability

The original contributions presented in the study are included in the article/Supplementary Material, and further inquiries can be directed to the corresponding authors.
